# Magnetic resonance imaging‐based radiomics nomogram for the evaluation of therapeutic responses to neoadjuvant chemohormonal therapy in high‐risk non‐metastatic prostate cancer

**DOI:** 10.1002/cam4.70001

**Published:** 2024-07-19

**Authors:** Xiao‐Hui Wu, Zhong‐Tian Ruan, Zhi‐Bin Ke, Fei Lin, Jia‐Yin Chen, Yu‐Ting Xue, Bin Lin, Shao‐Hao Chen, Dong‐Ning Chen, Qing‐Shui Zheng, Xue‐Yi Xue, Yong Wei, Ning Xu

**Affiliations:** ^1^ Department of Urology, Urology Research Institute, The First Affiliated Hospital Fujian Medical University Fuzhou China; ^2^ Department of Urology, National Region Medical Center, Binhai Campus of the First Affiliated Hospital Fujian Medical University Fuzhou China; ^3^ Fujian Key Laboratory of Precision Medicine for Cancer, The First Affiliated Hospital Fujian Medical University Fuzhou China

**Keywords:** neoadjuvant chemohormonal therapy, nomogram, pathological responses, prostate cancer, radiomics

## Abstract

**Purpose:**

The aim of this study was to assess the potential application of a radiomics features‐based nomogram for predicting therapeutic responses to neoadjuvant chemohormonal therapy (NCHT) in patients with high‐risk non‐metastatic prostate cancer (PCa).

**Methods:**

Clinicopathologic information was retrospectively collected from 162 patients with high‐risk non‐metastatic PCa receiving NCHT and radical prostatectomy at our center. The postoperative pathological findings were used as the gold standard for evaluating the efficacy of NCHT. The least absolute shrinkage and selection operator (LASSO) was conducted to develop radiomics signature. Multivariate logistic regression analyses were conducted to identify the predictors of a positive pathological response to NCHT, and a nomogram was constructed based on these predictors.

**Results:**

Sixty‐three patients (38.89%) experienced positive pathological response to NCHT. Receiver operating characteristic analyses showed that the area under the curve (AUC) of periprostatic fat (PPF) radiomics signature was 0.835 (95% CI, 0.754–0.898), while the AUC of intratumoral radiomics signature was 0.822 (95% CI, 0.739–0.888). Multivariate logistic regression analysis revealed that PSA level, PPF radiomics signature and intratumoral radiomics signature were independent predictors of positive pathological response. A nomogram based on these three predictors was constructed. The AUC was 0.908 (95% CI, 0.839–0.954). The Hosmer–Lemeshow goodness‐of‐fit test showed that the nomogram was well calibrated. Decision curve analysis revealed the favorable clinical practicability of the nomogram. The nomogram was successfully validated in the validation cohort. Kaplan–Meier analyses showed that nomogram and positive pathological response were significantly related with survival of PCa.

**Conclusion:**

The radiomics–clinical nomogram based on mpMRI radiomics features exhibited superior predictive ability for positive pathological response to NCHT in high‐risk non‐metastatic PCa.

## 1 INTRODUCTION

Prostate cancer (PCa) is one of the prevailing malignant tumors of the genitourinary system in males worldwide, with its morbidity in men rising to second place, being the sixth leading cause of death.^1^ According to the European Association of Urology (EAU) guidelines, localized PCa is categorized into low‐risk, intermediate‐risk, and high‐risk groups.^2^ High‐risk PCa represents a risk classification characterized by a heightened potential for biochemical recurrence (BCR), metastatic progression, and cancer‐related mortality, accounting for 15%–20% of clinically localized PCa cases.^3^ Neoadjuvant hormonal therapy (NHT) before radical prostatectomy (RP) has shown significant improvement in pathological outcomes, but it has not been found to provide a survival benefit for high‐risk PCa.^4^ Several studies have proved that neoadjuvant chemohormonal therapy (NCHT) can provide positive pathological benefit, and also was associated with prolonged biochemical recurrence‐free survival (bRFS) and overall survival (OS).^5^, ^6^, ^7^

Previous studies have suggested several strategies for identifying positive pathological response to NCHT among PCa patients, including prostate‐specific antigen (PSA) dynamics and prostate‐specific membrane antigen positron emission tomography/computed tomography (PSMA PET/CT).^8^, ^9^ However, these methods primarily focused on a single modality, lacked quantifiable risk measures, and exhibited limited accuracy. Radiomics, encompassing the retrieval of extractable high‐dimensional information from digital images, has the potential to furnish nonvisual insights pertaining to tumor heterogeneity and the fundamental pathophysiology.^10^ Previous studies have demonstrated the significant value of radiomic features extracted from multiparametric magnetic resonance imaging (mpMRI) in detecting PCa,^11^ discriminating Gleason score,^12^ predicting BCR status,^13^ and assessing treatment response.^14^ However, there is currently a lack of reports investigating the use of radiomic features to evaluate treatment responses to NCHT in patients with high‐risk non‐metastatic PCa.

Accordingly, the present study aimed to investigate the prostate mpMRI radiomics features associated with NCHT treatment responses, and to evaluate the potential application of radiomics features‐based nomogram in predicting the therapeutic responses to NCHT in patients with high‐risk non‐metastatic PCa.

## 2 MATERIALS AND METHODS

### 2.1 Patients and follow‐up

This study obtained approval from the Ethics Committee of the First Affiliated Hospital of Fujian Medical University. Written informed consent was secured from all patients who took part in the study. We conducted a retrospective collection of clinicopathological data from 162 patients diagnosed with high‐risk non‐metastatic PCa who underwent NCHT followed by RP at our center between January 2016 and January 2022. Before initiating NCHT, all cases received a pathological diagnosis of prostate adenocarcinoma via prostate biopsy. The 2017 Tumor, Node, Metastasis (TNM) classification guidelines were applied for staging PCa. High‐risk PCa, according to the 2021 EAU guidelines, encompasses localized high‐risk PCa (PSA >20 ng/mL, International Society of Urological Pathology (ISUP) grade 4/5, or cT2c stage) and locally advanced PCa (cT3‐4 stage or cN+ regardless of any PSA and ISUP grade). The absence of distant metastasis was confirmed through pretreatment computed tomography (CT), bone scanning, or PSMA PET/CT.

The inclusion criteria were as follows: (1) biopsy‐proven PCa; (2) patients underwent prostate mpMRI before commencing NCHT. The exclusion criteria were as follows: (1) were without T2 weighted images (T2WI) or apparent‐diffusion coefficient (ADC) images [*n* = 7]; (2) incomplete clinicopathologic data [*n* = 3]; (3) mpMRI images were of insufficient quality [*n* = 12]; (4) receiving any previous anticancer therapy [*n* = 4]; and (5) previous occurrence of anaphylactic reactions to chemotherapeutic medications or contrast agents [*n* = 3]. After excluding 29 patients who met the above exclusion criteria, the final analysis included 162 participants with complete clinicopathologic data, with 113 cases in the training group and 49 cases in the validation group.

Routine follow‐up for patients was conducted through various means, including In‐person visits, telephone communication. During follow‐up visits, PSA levels are monitored, and CT scans of the chest, abdomen, and pelvis, as well as MRI of the pelvis, and bone scanning are performed.

### 2.2 NCHT protocols and therapeutic response assessment

The NCHT treatment protocols were as follows^15^, ^19^: (1) intravenous infusion of docetaxel was given at a dose of 75 mg/m^2^ with a 21‐day cycle, along with oral prednisone administered twice daily at a dose of 5 mg; (2) subcutaneous injection of 3.6 mg of goserelin/leuprorelin, plus oral administration bicalutamide 50 mg/d with a cycle of 28 days. Each enrolled patient received 4–6 cycles of neoadjuvant chemotherapy (NACT) treatment before surgery, and skilled surgeons conducted RP in addition to standard pelvic lymph node dissection (PLND) within 3–4 weeks following NCHT.

The postoperative pathological findings were used as the gold standard for evaluating the efficacy of NCHT. The pathological complete remission (pCR) was defined as reduced glandular volume, decreased glandular density, increased periglandular density, and almost complete degeneration of cancer cells.^16^ The minimal residual disease (MRD) was defined as a maximum cross‐sectional size of the residual lesion less than 5 mm, whereas the significant residual disease (SRD) was defined as a maximum cross‐sectional size of the residual lesion greater than 5 mm. The pCR and MRD were assigned to positive pathological response, while the SRD assigned to unfavorable pathological response.^7^

### 2.3 Examination procedure of mpMRI

All patients underwent mpMRI (SIEMENS Verio 3.0 T) examination within 2 weeks before NACT. A supine position was adopted for the patient, and a comprehensive scan of the entire prostate was executed with the scanning range centered on the central part of the prostate. The patient was asked to have a bowel movement before the examination and ensure moderate bladder filling. The scanning sequences included transverse, sagittal, and frontal T2WI images, diffusion‐weighted imaging (DWI) images, and corresponding ADC maps. The detailed mpMRI sequences parameters were presented in Table S1. The gadopentetate dimeglumine (Gd‐DTPA) was administered via a pressure injector into the dorsal hand vein at a flow rate of 3 mL/s. A total of 18 sequential scans were performed, with each individual scan lasting for 11 s.

### 2.4 Periprostatic fat and intratumoral area segmentation

**FIGURE 1:**
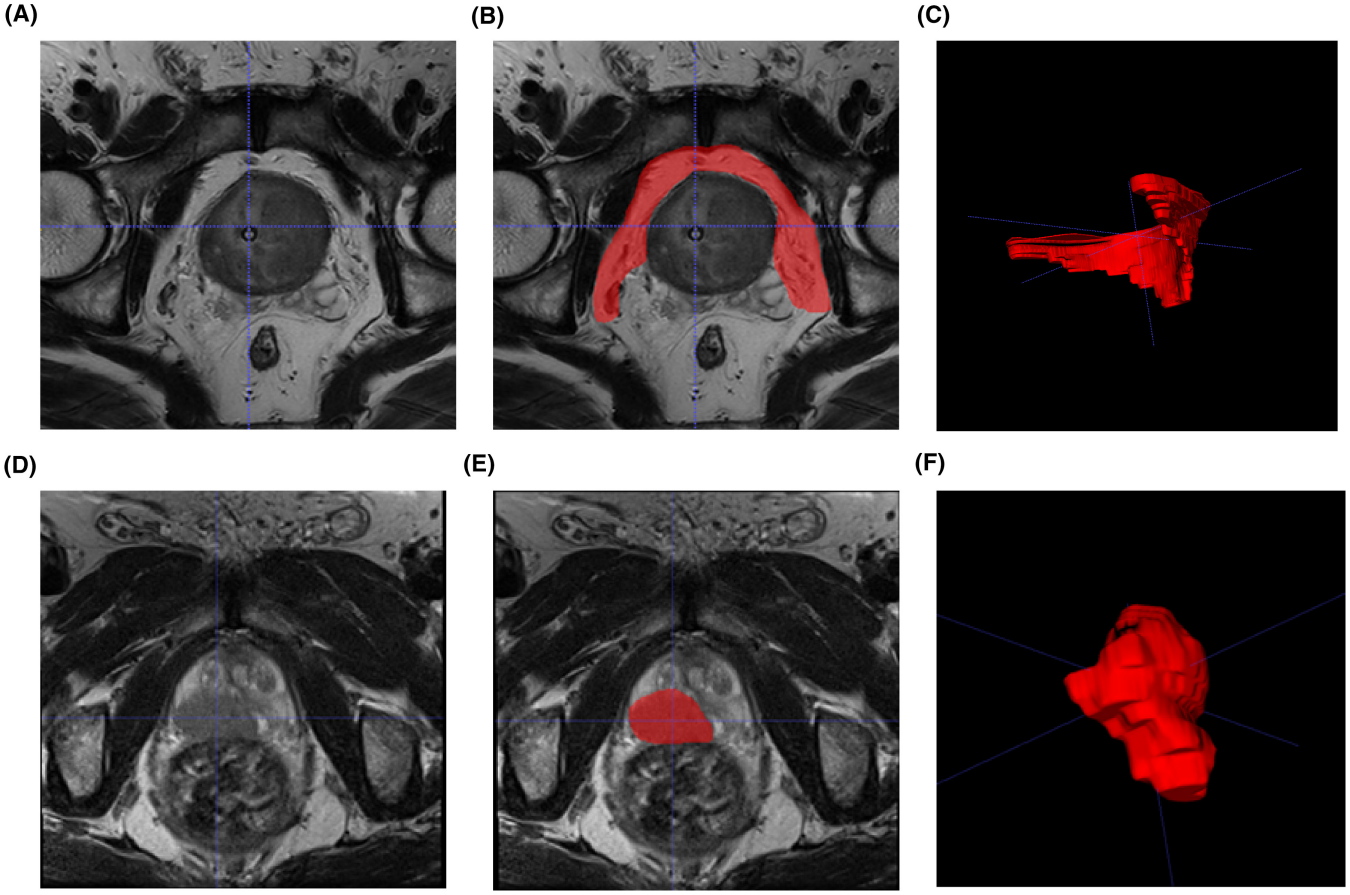
Schematic diagram of PPF and intratumoral ROI in MRI images of prostate cancer patients. (A) T2WI sequence showing prostate cancer in the PPF region. (B) Outline of the PPF ROI in the T2WI sequence. (C) Generated PPF ROI in the T2WI sequence. (D) T2WI sequence showing prostate cancer in the intratumoral region. (E) Outline of the intratumoral ROI in the T2WI sequence. (F) Generated intratumoral ROI in the T2WI sequence. ADC, apparent‐diffusion coefficient; MRI, magnetic resonance imaging; PCa, prostate cancer; PPF, periprostatic fat; ROI, regions of interest; T2WI, T2‐weighted imaging.

Two expert radiologists, each having more than 8 years of expertise in interpreting prostate mpMRI, blinded to each other's delineations and NCHT treatment response information, independently outlined the regions of interest (ROIs) for periprostatic fat (PPF) and intratumoral areas in T2WI and ADC images performing ITK‐SNAP software version 3.6.0. (Yushkevich P and Gerig G). The PPF region includes the neurovascular bundles, where the seminal vesicle and metastatic lymph nodes excluded. The PPF and intratumoral ROIs were showed in Figure 1. To select robust features, 50 patients were randomly selected to conduct a test–retest study. The inter‐observer repeatability of the extracted features between two radiologists was assessed, and the intra‐observer repeatability was assessed by comparing the extracted features of the same radiologist (twice, 1 week apart).

### 2.5 Extraction of radiomics features

Radiomics features were extracted using Python (version 3.7.3) package PyRadiomics version 3.0. Radiomic features were obtained from the original images, in addition to applying two basic image filters, which were Laplacian of Gaussian (LoG) and wavelet images. The extracted features included: (1) shape features; (2) first‐order statistical features; (3) gray‐level run length matrix (GLRLM) features; (4) gray‐level co‐occurrence matrix (GLCM) features; (5) gray‐level dependence matrix (GLDM) features; (6) gray‐level size zone matrix (GLSZM) features; and (7) neighboring gray tone difference matrix (NGTDM) features.

### 2.6 Construction of radiomics signatures

To remove scale variations and ensure comparability, all features underwent normalization using the *Z*‐score transform. Subsequently, features with low repeatability were excluded from further analysis. Inter‐observer and intra‐observer repeatability were analyzed through the use of the intraclass correlation coefficient (ICC) (using R package “psych” version 2.4.3). We employed a threshold of ICC >0.8 to select features for further investigation. The least absolute shrinkage and selection operator (LASSO) method (using R package “glmnet” version 4.1–7 and “pROC” version 1.18.0) was applied to identify the most reliable predictive radiomics features that demonstrated excellent reproducibility and strong association with positive pathological response to NCHT. The selected radiomics features were analyzed by employing logistic risk regression to develop PPF and intratumoral radiomics signatures.

### 2.7 Construction and validation of radiomics–clinical nomogram

Univariate logistic regression analysis was used to determine the correlation between radiomics signatures, clinical characteristics, and positive pathological response. Subsequently, multivariate logistic regression analysis was performed to identify independent predictors of a positive pathological response to NCHT. Based on these potential predictors, a radiomics–clinical nomogram was constructed. The diagnostic performance of the nomogram was evaluated using sensitivity, specificity, positive predictive value (PPV), negative predictive value (NPV), and accuracy calculations. Additionally, the nomogram's performance was further assessed by constructing receiver operating characteristic (ROC) curve. DeLong's test was employed to compare the area under the curve (AUC) of the radiomics–clinical nomogram with different predictors.^17^ Furthermore, the HosmerLemeshow test and calibration plots were utilized to examine the nomogram's calibration.^18^ To evaluate the clinical utility of the nomogram, decision‐curve analysis (DCA) was conducted.^19^ Finally, Kaplan–Meier analysis was used to investigate the relationship between the nomogram and the survival of patients with PCa. The radiomics–clinical nomogram was constructed and validated with the aid of R packages including “Hmisc” package version 5.0‐1, “car” package version 3.1‐2, “rms” package version 6.6‐0, “pROC” package version 1.18‐0, “survival” package version 3.5‐5, “survminer” package version 0.4.9, and “rmda” package version 1.6.

### 2.8 Statistical analyses

Statistical analyses were performed using SPSS version 26 (IBM SPSS, Inc., Armonk, NY) and R software (R Foundation for Statistical Computing, Vienna, Austria version 4.1.0). For categorical variables, comparisons were made using the chi‐square test or Fisher's exact test, while continuous variables were assessed using the independent *t*‐test or Mann–Whitney *U*‐test. Kaplan–Meier analysis was conducted to investigate the association between positive pathological response to NCHT and the survival of patients with PCa. A two‐sided *p* < 0.05 was considered statistically significant.

## 3 RESULTS

### 3.1 Baseline characteristics

**FIGURE 2:**
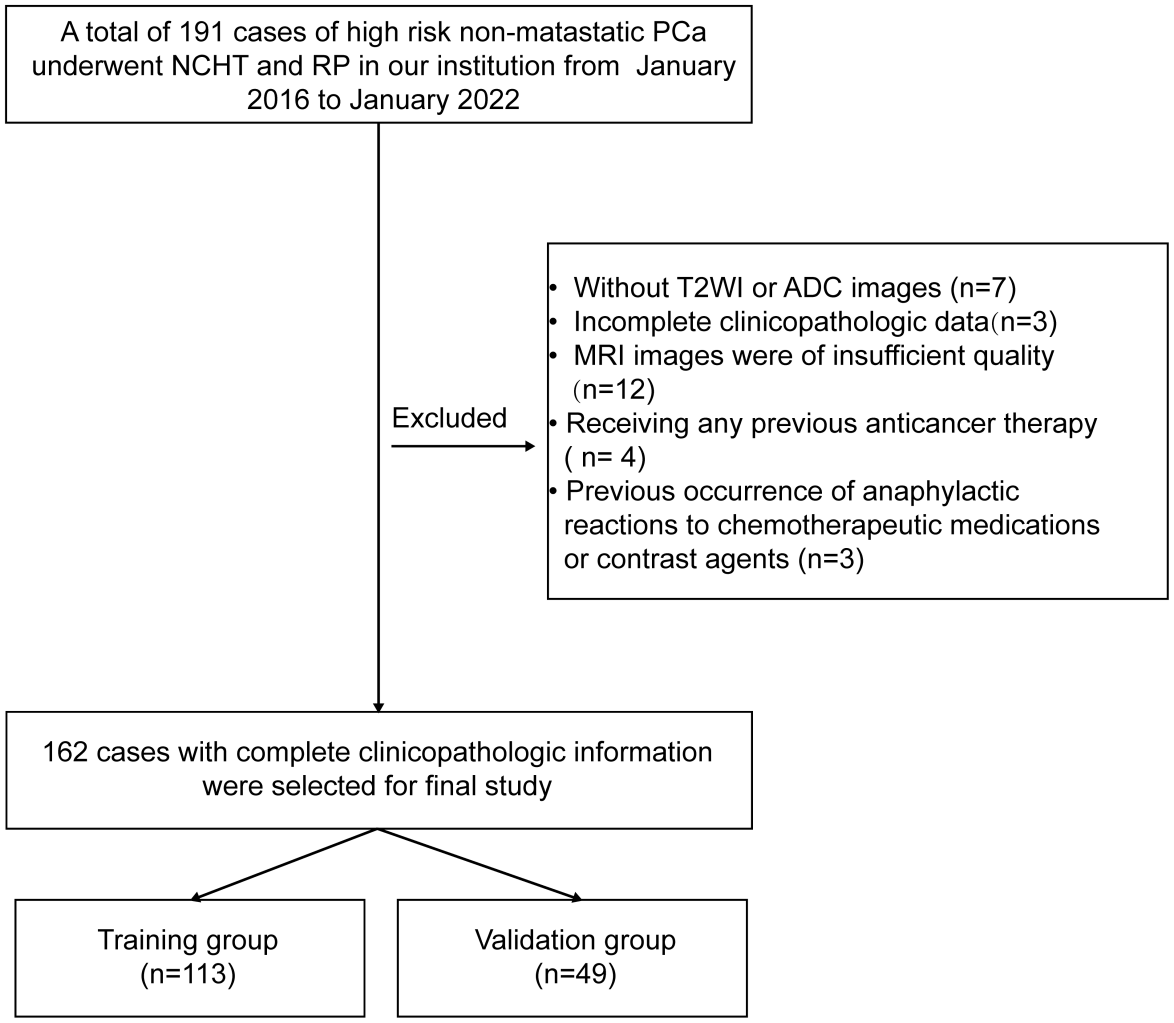
Flow diagram of the study.

The flow diagram of this study is presented in Figure 2. The baseline clinical characteristics information of 162 high‐risk non‐metastatic PCa patients was listed in Table 1. The number of patients who experienced positive pathological response to NCHT (pCR and MRD) was 63 (38.89%), and the number of patients who experienced negative response to NCHT (SRD) is 99 (61.11%). There were no significant differences observed in initial PSA level, ISUP grading group of biopsy specimens, EAU clinical T stage, PI‐RADS v2 score, age, BMI as well as the positive pathological response between the training group and validation group.

**TABLE 1:** Clinical characteristics of 162 patients with high‐risk non‐metastatic prostate cancer in the training and validation cohorts.

Characteristics	Whole cohort (*n* = 162)	Training cohort (*n* = 113)	Validation cohort (*n* = 49)	*p*‐value
Age (years), mean ± SD	70.28 ± 6.21	69.97 ± 6.62	71.02 ± 5.15	0.276
BMI (kg/m^2^), mean ± SD	23.38 ± 3.69	23.62 ± 3.77	22.82 ± 3.46	0.212
Initial PSA at diagnosis (ng/mL), median (range)	29.81 (17.24–86.11)	29.49 (17.24–86.11)	30.82 (18.39–77.77)	0.099
ISUP grading group of biopsy specimens, *n* (%)				
3	46 (28.40%)	34 (30.09%)	12 (24.49%)	0.721
4	75 (46.30%)	52 (46.02%)	23 (46.94%)	
5	41 (25.30%)	27 (23.89%)	14 (28.57%)	
EAU clinical T stage, *n* (%)				
2c	12 (7.41%)	10 (8.85%)	2 (4.08%)	0.675
3a	63 (38.89%)	45 (39.82%)	18 (36.73%)	
3b	76 (46.91%)	50 (44.25%)	26 (53.06%)	
4	11 (6.79%)	8 (7.08%)	3 (6.12%)	
PI‐RADS v2 score, *n* (%)				
3	16 (9.88%)	11 (9.73%)	5 (10.20%)	0.597
4	79 (48.77%)	58 (51.33%)	21 (42.86%)	
5	67 (41.36%)	44 (38.94%)	23 (46.94%)	
Pathological response, *n* (%)				
pCR	16 (9.88%)	10 (8.85%)	6 (12.24%)	0.935
MRD	47 (29.01%)	34 (30.09%)	13 (26.53%)	
SRD	99 (61.11%)	69 (61.06%)	30 (61.22%)	

Abbreviations: BMI, body mass index; EAU, European Association for Urology; ISUP, International Society of Urological Pathology; PI‐RADS v2, Prostate Imaging Reporting and Data System version 2; PSA, prostate‐specific antigen.

**FIGURE 3:**
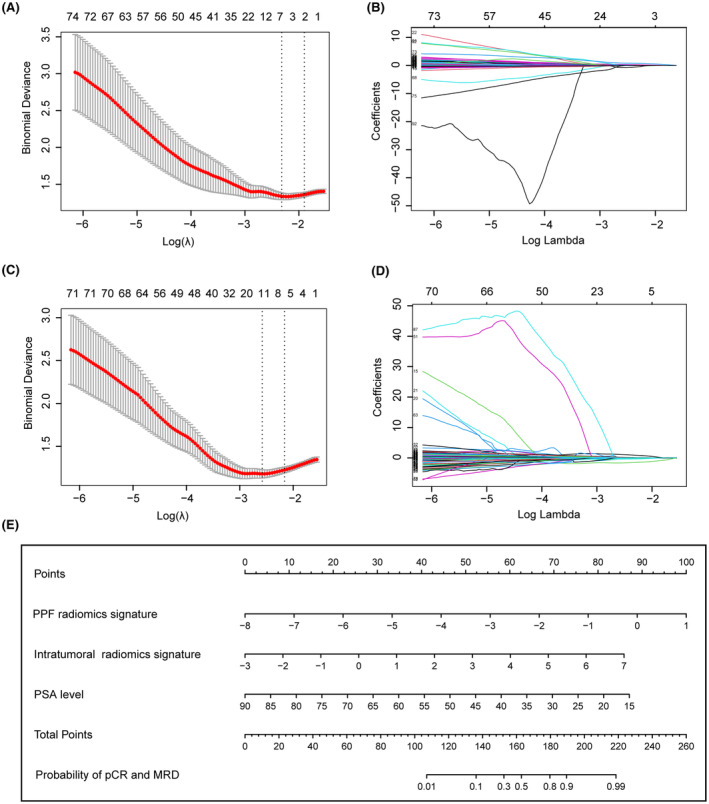
Construction of radiomics signatures and radiomics‐clinical nomogram. (A, C) Partial likelihood deviance plotted against log (λ). The *y*‐axis represents the partial likelihood deviance, while the lower *x*‐axis indicates log (λ), and the upper *x*‐axis represents the average number of predictors. Dotted vertical lines indicate the optimal values determined by the minimum criteria and 1 standard error of the minimum criteria. The tuning parameter (λ) was selected in the LASSO model via 10‐fold cross‐validation based on minimum criteria. (B) Six features with nonzero coefficients were selected to build the PPF radiomics signature. (D) Six features with nonzero coefficients were selected to build the intratumoral radiomics signature. (E) The nomogram for predicting positive pathological response to NCHT. LASSO, least absolute shrinkage and selection operator; NCHT, neoadjuvant chemohormonal therapy; PPF, periprostatic fat.

**FIGURE 4:**
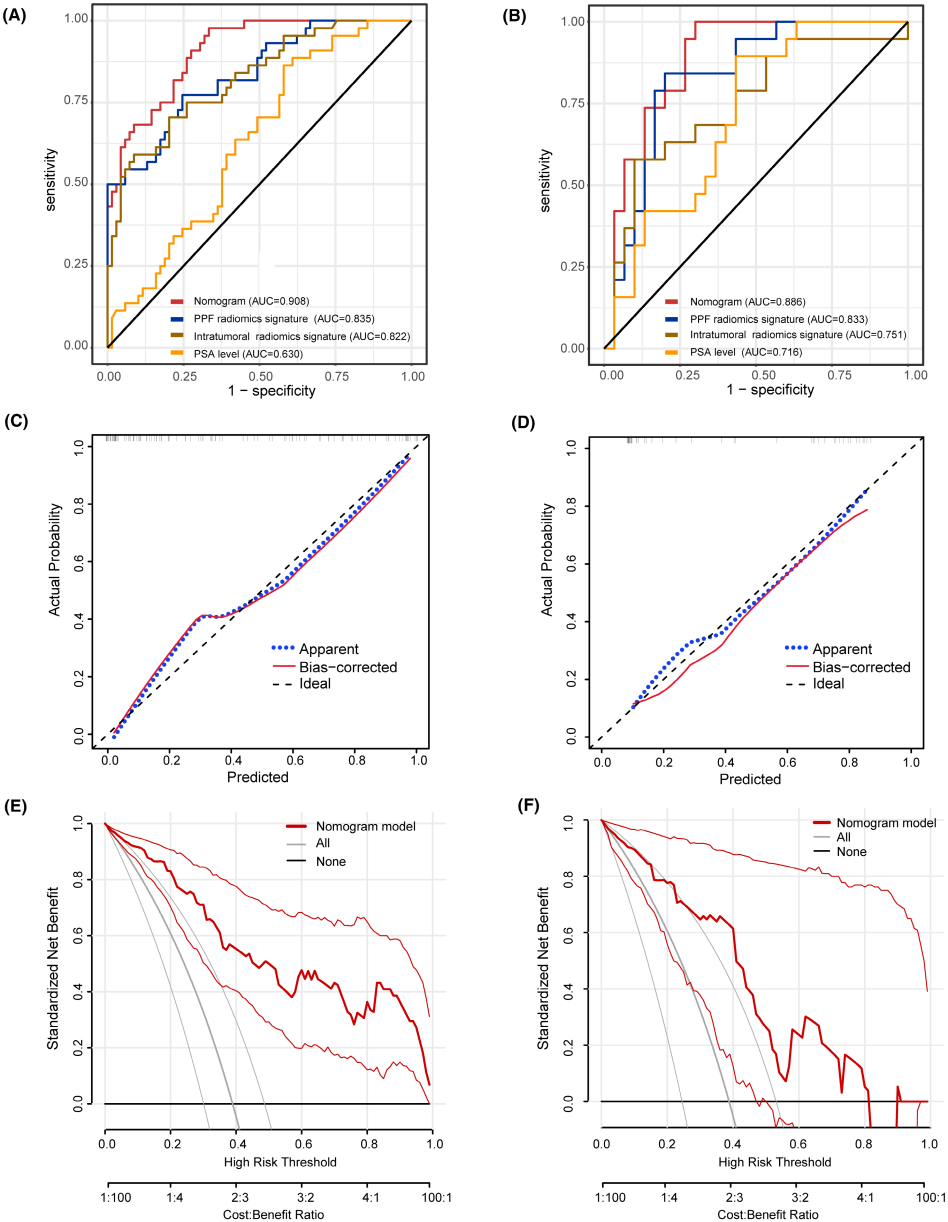
Validation of the radiomics‐clinical nomogram. (A, B) Receiver operating characteristic curve analysis of the nomogram and three independent predictors. (C, D) Hosmer–Lemeshow goodness‐of‐fit test was used to evaluate model calibration in both the training group (*χ* ^2^ = 8.843, *p* = 0.356) and the validation group (*χ* ^2^ = 5.200, *p* = 0.736). (E, F) Decision curve analysis demonstrated that the nomogram can facilitate clinical decision‐making within a considerable risk threshold.

**FIGURE 5:**
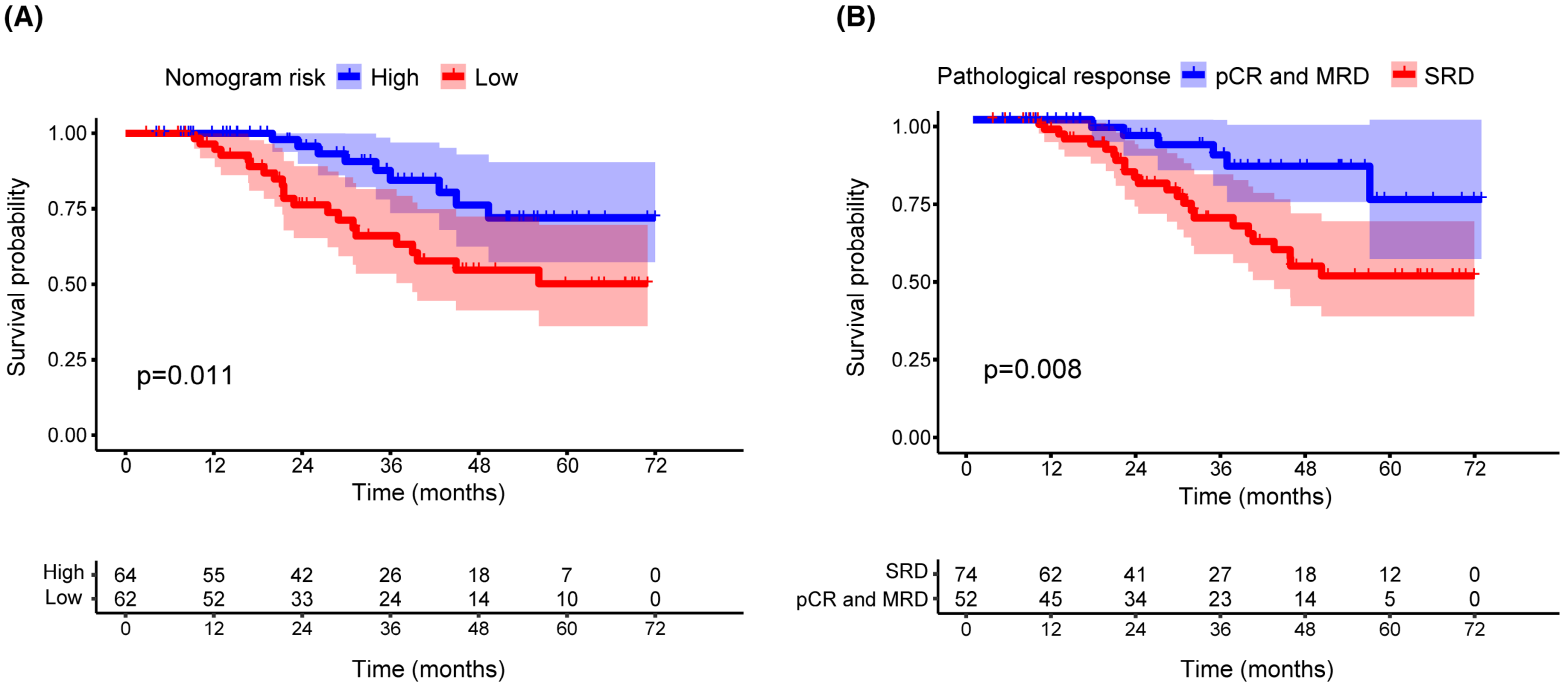
Kaplan–Meier curve analyses for PFS of the radiomics‐clinical nomogram (A) and (B) positive pathological response to NCHT. NCHT, neoadjuvant chemohormonal therapy; PFS, progression‐free survival.

### 3.2 Identification of radiomics features and construction of radiomics signatures

We extracted 2632 radiomics features form the PPF ROI (1316 radiomics features from ADC and 1316 radiomics features from T2WI) and intratumoral ROI (1316 radiomics features from ADC and 1316 radiomics features from T2WI), respectively. Features with low reproducibility, indicated by intra‐ or inter‐observer ICC values below 0.8 were excluded from the analysis, the count of PPF features decreased to 1819 (895 features from ADC and 924 features from T2WI), while the count of intratumoral features decreased to 1784 (869 features from ADC and 915 features from T2WI). Subsequently, six radiomics features were obtained from PPF and six features were obtained from intratumoral area based on LASSO regression method (Table 2). Finally, logistic risk regression method was used to construct PPF radiomics signature (Figure 3A,B) and intratumoral radiomics signature (Figure 3C,D). The ROC analysis showed that the AUC of PPF radiomics signature was 0.835 (95% CI, 0.754–0.898) and 0.833 (95% CI, 0.699–0.924), while the AUC of intratumoral radiomics signature was 0.822 (95% CI, 0.739–0.888) and 0.751 (95% CI, 0.607–0.863) in the training group and validation group, respectively. Furthermore, the sensitivity, specificity, and accuracy of PPF and intratumoral radiomics signatures to predict positive pathological response to NCHT were calculated and presented in Table 4. These results demonstrated that PPF and intratumoral radiomics signatures possessed remarkable performance in predicting positive pathological response to NCHT.

**TABLE 2:** Summary of radiomic features of PPF and intratumoral radiomics signatures.

Radiomics signatures	MRI sequences	Radiomics features	Coef.
PPF radiomics signature	T2WI	Wavelet.HLH_glcm_MCC	−2.948
	T2WI	Wavelet.HLL_glcm_Correlation	−0.354
	T2WI	Wavelet.LLH_glszm_SizeZoneNonUniformityNormalized	−0.981
	T2WI	Wavelet.HHL_glcm_Correlation	−1.045
	T2WI	Wavelet.LLH_gldm_LargeDependenceLowGrayLevelEmphasis	2.223
	ADC	Original_ngtdm_Busyness	−0.756
Intratumoral radiomics signature	T2WI	Original_shape_Elongation	1.443
	T2WI	Wavelet.LLH_glcm_Correlation	0.987
	ADC	Wavelet.HHL_glszm_SmallAreaHighGrayLevelEmphasis	0.918
	T2WI	Original_shape_MinorAxisLength	1.426
	T2WI	Wavelet.LHH_firstorder_InterquartileRange	−0.380
	T2WI	Wavelet.LLH_glszm_SizeZoneNonUniformityNormalized	−2.589

Abbreviations: ADC, apparent‐diffusion coefficient; Coef, coefficient; GLCM, gray‐level co‐occurrence matrix; GLSZM, gray‐level size zone matrix; MRI, magnetic resonance imaging; PPF, periprostatic fat; NGTDM, neighboring gray tone difference matrix; T2WI, T2‐weighted imaging.

### 3.3 Construction and validation of radiomics–clinical nomogram

Univariate logistic regression analysis showed that initial PSA level at diagnosis (*p* = 0.008), PPF radiomics signature (*p* < 0.001) and intratumoral radiomics signature (*p* < 0.001) were associated with a positive pathological response to NCHT. Subsequently, multivariate logistic regression analysis demonstrated that the initial PSA level at diagnosis (*p* = 0.008), PPF radiomics signature (*p* < 0.001), and intratumoral radiomics signature (*p* = 0.002) were independent predictors of a positive pathological response to NCHT (Table 3). Based on these three independent factors, a nomogram for predicting positive pathological response to NCHT was developed (Figure 3E). As showed in Table 4, the sensitivity, specificity and accuracy to predict positive response to NCHT was 0.727, 0.855 and 0.805 in the training group respectively. ROC analysis demonstrated that the nomogram had an AUC of 0.908 (95% CI, 0.839–0.954), which significantly outperformed the PPF radiomics signature (AUC, 0.835, 95% CI, 0.754–0.898, Delong test, *z* = 2.379, *p* = 0.017), intratumoral radiomics signature (AUC, 0.822, 95% CI, 0.739–0.888, Delong test, *z* = 2.762, *p* = 0.006), and PSA level (AUC, 0.630, 95% CI, 0.534–0.719, Delong test, *z* = 5.114, *p* < 0.001) (Figure 4A, Table 5). The Hosmer–Lemeshow goodness‐of‐fit test indicated that the nomogram was well calibrated (*χ* ^2^ = 8.843, *p* = 0.356) (Figure 4C). To further assess the practical utility of nomogram in clinical decision‐making, we quantified the net benefit of decision thresholds at various probability levels using DCA. As illustrated in Figure 4E,F, the “All” curve represented the scenario where clinical interventions were applied to all patients, while the “None” curve signified the absence of clinical interventions for all patients. The red curve corresponded to the decision curve of the nomogram model. The net benefit of nomogram curve was higher than that of “All” curve and “None” curve when the threshold probability was 0–1.0, indicating that nomogram has high clinical practicability within the threshold probability range of 0–1.0 (Figure 4E).

**TABLE 3:** Univariate and multivariate logistic regression analyses exploring independent predictors of positive pathological response to neoadjuvant chemohormonal therapy.

Variables	Positive pathological responses				
	Univariate		Multivariate		
	OR (95% CI)	*p*‐value	*β*	OR (95% CI)	*p*‐value
Age (years)	1.000 (0.944–1.059)	0.990			
BMI (kg/m^2^)	1.108 (0.997–1.232)	0.056			
Initial PSA at diagnosis (ng/mL)	0.947 (0.909–0.986)	0.008	−0.096	0.909 (0.847–0.975)	0.008
ISUP grading group of biopsy specimens					
4 versus 3	1.048 (0.436–2.518)	0.917			
5 versus 3	0.602 (0.206–1.757)	0.353			
EAU clinical T stage					
3a versus 2c	0.552 (0.139–2.197)	0.399			
3b versus 2c	0.613 (0.157–0.239)	0.482			
4 versus 2c	1.000 (0.156–6.420)	1.000			
PI‐RADS v2 score					
4 versus 3	0.585 (0.158–2.161)	0.421			
5 versus 3	1.000 (0.265–3.769)	1.000			
PPF radiomics signature	2.718 (1.866–3.960)	<0.001	0.916	2.499 (1.545–4.042)	<0.001
Intratumoral radiomics signature	2.718 (1.782–4.145)	<0.001	0.708	2.030 (1.284–3.209)	0.002

Abbreviations: BMI, body mass index; CI, confidence interval; ISUP, International Society of Urological Pathology; OR, odds ratio; PI‐RADS v2, Prostate Imaging Reporting and Data System version 2; PPF, periprostatic fat; PSA, prostate‐specific antigen.

**TABLE 4:** Summary of models' performance in the training and validation cohorts.

Cohorts	Models	AUC (95% CI)	Sensitivity	Specificity	PPV	NPV	Accuracy
Training cohort	Nomogram	0.908 (0.839–0.954)	0.727	0.855	0.762	0.831	0.805
	PPF radiomics signature	0.835 (0.754–0.898)	0.704	0.797	0.689	0.809	0.761
	Intratumoral radiomics signature	0.822 (0.739–0.888)	0.591	0.841	0.703	0.763	0.743
Validation cohort	Nomogram	0.886 (0.763–0.959)	0.737	0.867	0.778	0.838	0.816
	PPF radiomics signature	0.833 (0.699–0.924)	0.684	0.833	0.722	0.806	0.775
	Intratumoral radiomics signature	0.751 (0.607–0.863)	0.526	0.900	0.769	0.750	0.755

Abbreviations: AUC, area under the receiver operating characteristic curve; CI, confidence interval; NPV, negative predictive value; PPF, periprostatic fat; PPV, positive predictive value.

**TABLE 5:** Effectiveness of nomogram and three features for differentiating the positive pathological response by ROC curve analysis in the training cohort.

Parameters	AUC	95%CI	DeLong test	
			*z* statistic	*p*‐value
Nomogram	0.908	0.839–0.954	–	–
PPF radiomics signature	0.835	0.754–0.898	2.379^a^	0.017
Intratumoral radiomics signature	0.822	0.739–0.888	2.762^b^	0.006
PSA level	0.630	0.534–0.719	5.114^c^	<0.001

Abbreviations: AUC, area under the receiver operating characteristic curve; CI, confidence interval; PPF, periprostatic fat; ROC, receiver operating characteristic curve.

^a^ Nomogram versus PPF radiomics signature in distinguishing the positive pathological response.

^b^ Nomogram versus Intratumoral radiomics signature in distinguishing the positive pathological response.

^c^ Nomogram versus PSA level in distinguishing the positive pathological response.

The validation of the nomogram in the validation group is demonstrated in Table 4. The sensitivity, specificity, and accuracy to predict a positive response were 0.737, 0.867, and 0.816, respectively. ROC analysis indicated that the nomogram's AUC was 0.886 (95% CI, 0.763–0.959), which significantly outperformed the AUC of the intratumoral radiomics signature (AUC, 0.751, 95% CI, 0.607–0.863, Delong test, *z* = 2.180, *p* = 0.030), and PSA level (AUC, 0.716, 95% CI, 0.569–0.835, Delong test, *z* = 2.144, *p* = 0.032) (Figure 4B, Table S2). The Hosmer–Lemeshow goodness‐of‐fit test indicated that the nomogram was well calibrated (*χ* ^2^ = 5.200, *p* = 0.736) (Figure 4D). The DCA showed that applying the clinical‐radiomics nomogram to inform clinical decisions would lead to superior outcomes in the threshold probability range of 0–0.8 in the validation group (Figure 4F).

### 3.4 Survival analysis

Among the 162 high‐risk non‐metastatic PCa patients, only 126 cases had complete follow‐up information. During the follow‐up, 9 patients died of tumor progression, 14 patients experienced BCR, and 7 patients progressed to metastasis. The median follow‐up duration in the study was 38.90 months (95% CI, 31.17–46.64 months). The higher nomogram risk score was significantly associated with longer progression‐free survival (PFS) compared to lower risk score (Figure 5A). In addition, Kaplan–Meier analysis showed that patients with positive pathological response to NCHT experienced significantly better PFS compared to patients with SRD (Figure 5B).

## 4 DISCUSSION

To the best of our knowledge, this study is the first to explore the potential application of a nomogram constructed based on mpMRI radiomics features for predicting the positive pathological response to NCHT in non‐metastatic high‐risk PCa patients. Our findings revealed that PPF and intratumoral radiomics signatures were significantly related with therapeutic responses to NCHT. Moreover, we constructed a nomogram for predicting positive pathological response to NCHT by combining three independent predictors: PPF radiomics signature, intratumoral radiomics signature, and PSA level. The ROC analysis revealed that the nomogram exhibited superior predictive performance compared to any individual predictor and the Hosmer–Lemeshow test revealed that the nomogram has good calibration. Furthermore, the DCA analysis provided evidence supporting the feasibility of utilizing the nomogram to facilitate beneficial clinical decision‐making.

In recent years, researchers have observed features in the adipose tissue surrounding tumors that are linked to tumor progression and response to neoadjuvant therapy, and these features can be captured and analyzed by radiomics methods. For example, Shaish et al. performed a study investigating the potential value of pretreatment MRI‐based radiomics features extracted from intratumor and the mesorectal compartment in predicting neoadjuvant treatment‐related outcomes for patients with locally advanced rectal cancer undergoing neoadjuvant chemoradiation. They found that the radiomics model combing the tumor and mesorectal features had robust accuracy in predicting pCR, tumor regression grade (TRG), and neoadjuvant rectal (NAR) score after neoadjuvant chemoradiation.^20^ Jayaprakasam et al. revealed that radiomics features derived from mesorectal fat exhibited predictive capabilities for pCR, local and distant recurrence, as well as post‐treatment T and N categories in patients with locally advanced rectal cancer.^21^ In our study, PPF radiomics signature was an independent predictor of positive pathological response to NCHT, and had steady predictive efficacy in predicting NCHT treatment responses. These findings provide compelling evidence of a substantial association between the efficacy of NCHT response and radiomic features of periprostatic adipose tissue surrounding tumors, suggesting the potential utility of the PPF radiomic signature in predicting a positive pathological response to NCHT.

Radiomics features encompass morphological features, textural features, and metabolic features. These radiomics features could reflect the different biological characteristics, metabolic activity, and pathological features of the periprostatic adipose and intratumoral tissue.^22^, ^23^ The associations between NCHT treatment responses and PPF radiomics features may be complicated. It is worth noting that radiomic features, being collected from the entire tissue, can capture tumor heterogeneity, which is well known to be closely associated with tumor progression and treatment resistance. Previous studies have pointed out that PPF thickness^24^ and volume^25^ were independent predictors of androgen deprivation therapy (ADT) efficacy in PCa patients. In addition, Abd Elmageed et al. demonstrated that PCa triggers pro‐tumorigenesis in periprostatic adipose tissue. Their findings unveiled that the medium utilized for culturing PCa cells triggered tumor‐like alterations in preadipocytes, encompassing epithelial–mesenchymal transition, genetic variability, and the formation of tumor‐like lesion in vivo.^26^ These microscopic changes, imperceptible to the naked eye, could hold crucial information regarding drug resistance. Besides, the structure and metabolic level of periprostatic adipose tissue have been found to be associated with the growth and spread of PCa.^27^, ^28^ Studies have demonstrated that PCa cells can derive nutrients and energy from periprostatic adipose tissue, promoting tumor growth and metastasis.^29^, ^30^ Coy et al. reported that lipid metabolism disturbances in periprostatic adipose tissue can influence tumor cell processes by inducing metabolic changes.^31^ The comprehensive analysis of these minute alterations and identification of the structure and metabolic changes feature in periprostatic adipose tissue through radiological methods may serve as a significant factor in predicting the effectiveness of NCHT treatment.

The administration of NCHT for non‐metastatic high‐risk PCa appears to be an effective neoadjuvant regimen, as it can lead to significant pathologic response and improved prognosis.^6^ Positive pathological response to neoadjuvant therapy has been proved to bring survival benefits to patients in many malignant tumors, including gastric cancer,^32^ rectal cancer,^33^ breast cancer^34^ and PCa.^6^ In our study, 63 patients achieved a positive pathological response and Kaplan–Meier curves demonstrated that patients with pCR and MRD were significantly associated with a better PFS. Overall, early prediction of the pathological response to NCHT holds great importance as it can guide treatment decisions, improve patient outcomes, and facilitate advancements in cancer research and treatment. Previous research has indicated that neoadjuvant treatment response was related to various molecular or characteristics factors. Zhu et al. proposed a molecular predictive signature consisting of 10 genes, which can identify distinct neoadjuvant therapy benefits for high‐risk non‐metastatic PCa.^35^ Fan et al. suggested that lower PSA level, lower expression level of AR and higher expression level of Ki‐67 were independent predictive factors for positive pathologic response to NCHT.^36^ However, the response to NCHT of PCa is complex, and these individual clinical and biological markers are challenging to reflect the true state of NCHT efficacy and lack necessary validation. Radiomics analyses begins with medical imaging collected as part of routine clinical practice, making it a non‐invasive procedure that does not require additional costs. Accumulating evidence revealed that MRI radiomics bears the potential to screen PCa, monitor tumoral metastatic status and predict survival probability.^37^ Furthermore, Abdollahi et al. showed that MRI radiomics features performed well in monitoring intensity‐modulated radiation therapy (IMRT) responses in patients with PCa.^14^ However, there is currently no reported research on utilizing radiomic features from periprostatic adipose and intratumoral tissue to evaluate the efficacy of neoadjuvant therapy in high‐risk non‐metastatic PCa patients. Our study, for the first time, established a new nomogram based on clinical characteristics and MRI radiomic features for predicting the response to NCHT in PCa patients. The ROC analysis and Hosmer–Lemeshow test indicated favorable discrimination and calibration of the nomogram. While a model boasting better discrimination and calibration theoretically enhances its utility as a guiding tool for clinical management, the efficacy of such statistical metrics becomes insufficient when assessing the model's capacity to enhance clinical decision‐making. DCA, as a statistical method, assesses the utility of a model in facilitating clinical decisions. A model is clinically valuable only when it has net benefit above the “All” curve and “None” curve within a specific threshold. In our study, the net benefit of nomogram curve is higher than that of “All” curve and “None” curve when the threshold probability is 0–1.0 in the training group, and 0–0.8 in the validation group, suggesting that this nomogram model can promote clinical decision‐making within a considerable risk threshold. Taken together, these results comprehensively indicated that the nomogram possessed a high predictive performance for NCHT efficacy and exhibited good clinical practicality.

This study has several limitations. First, this was a retrospective study conducted at a single center, and because of the relatively low number of high‐risk non‐metastatic PCa patients undergoing NCHT, the available sample size for research is limited. Future prospective validation in multicenter and large‐scale cohorts is necessary. Second, despite the manual segmentation of the ROIs performed by two radiologists, complete elimination of their subjective bias was not possible. Hence, several measures were implemented to mitigate potential deviations, including blinding to the postoperative pathology results of patients, as well as calculating intra‐ or inter‐observer ICC to select features, thereby reducing the extent of bias. Thirdly, the follow‐up period in this study was relatively short. Therefore, longer‐term follow‐up investigations are necessary in the future to further elucidate the correlation between the radiomic features and survival of PCa patients.

## 5 CONCLUSIONS

This study demonstrated that MRI radiomics features played an important role in evaluating the NCHT treatment efficacy for high‐risk non‐metastatic PCa. We identified three independent predictors (including: PPF radiomics signature, intratumoral radiomics signature, and initial PSA level) for positive pathological response to NCHT in high‐risk non‐metastatic PCa. The nomogram developed based on these three independent predictors exhibited a high predictive performance and demonstrated good clinical practicability. Furthermore, both the nomogram and positive pathological response to NCHT were significantly associated with survival of PCa patients.

## AUTHOR CONTRIBUTIONS

**Xiao‐Hui Wu:** Formal analysis (equal); methodology (equal); visualization (equal); writing – original draft (equal). **Zhong‐Tian Ruan:** Writing – original draft (equal). **Zhi‐Bin Ke:** Writing – original draft (equal). **Fei Lin:** Methodology (equal). **Jia‐Yin Chen:** Methodology (equal). **Yu‐Ting Xue:** Formal analysis (equal). **Bin Lin:** Formal analysis (equal). **Shao‐Hao Chen:** Data curation (equal). **Dong‐Ning Chen:** Data curation (equal). **Qing‐Shui Zheng:** Conceptualization (equal). **Xue‐Yi Xue:** Conceptualization (equal). **Yong Wei:** Project administration (equal); visualization (equal); writing – review and editing (equal). **Ning Xu:** Project administration (equal); writing – review and editing (equal).

## FUNDING INFORMATION

The study was supported by the "Eyas Plan" Youth Top ‐ notch Talent Project of Fujian Province (Grant number: SCYJHBJRC ‐ XN2021), Science and Technology Innovation Joint Fund project of Fujian province (Grant number: 2021Y9126 and 2023Y9078).

## CONFLICT OF INTEREST STATEMENT

All authors declare no conflict of interests.

## CONSENT

Not applicable.

## Supporting information


Table S1.

Table S2.

## Data Availability

The datasets used and/or analyzed during the current study are available from the corresponding author on reasonable request.
