# Supplementary material for: Magnetic resonance imaging‐based radiomics nomogram for the evaluation of therapeutic responses to neoadjuvant chemohormonal therapy in high‐risk non‐metastatic prostate cancer
Source: Cancer Med. 2024 Jul 19;13(14):e70001. doi: 10.1002/cam4.70001 (PMC11258568; doi:10.1002/cam4.70001)
Supplement: Supplementary file 1 — Table S1. [file CAM4-13-e70001-s001.docx]

| **Supplementary Table 1. Detailed sequence parameters for prostate mpMRI** | | | |
| --- | --- | --- | --- |
| Parameters | T2WI | T1W1 | DWI |
| TR/TE (ms) | 3500/101 | 700/11 | 3500/90 |
| Layer thickness (mm) | 3 | 3 | 3.5 |
| Layer spacing (mm) | 0.3 | 0.3 | 0.3 |
| Field of view (mm × mm) | 200×200 | 200×200 | 250×250 |
| Matrix size | 256×256 | 192×116 | 192×192 |
| Other | - | - | b values =0 and 1000 s/mm2 |
| T2WI, T2 weighted images; T1W1, T1 weighted images; DWI, diffusion-weighted imaging; mpMRI, multiparametric magnetic resonance imaging; TR, Repetition time; TE, Time echo | | | |
