# Supplementary material for: Magnetic resonance imaging‐based radiomics nomogram for the evaluation of therapeutic responses to neoadjuvant chemohormonal therapy in high‐risk non‐metastatic prostate cancer
Source: Cancer Med. 2024 Jul 19;13(14):e70001. doi: 10.1002/cam4.70001 (PMC11258568; doi:10.1002/cam4.70001)
Supplement: Supplementary file 2 — Table S2. [file CAM4-13-e70001-s002.docx]

| **Supplementary Table 2. Effectiveness of nomogram and three features for differentiating the positive pathological response by ROC curve analysis in the validation cohort** | | | | |
| --- | --- | --- | --- | --- |
| Parameters | AUC | 95%CI | DeLong test | |
|  |  |  | z statistic | p-value |
| Nomogram | 0.886 | 0.763-0.959 | - | - |
| PPF radiomics signature | 0.833 | 0.699-0.924 | 0.983^a^ | 0.326 |
| Intratumoral radiomics signature | 0.751 | 0.607-0.863 | 2.180^b^ | 0.030 |
| PSA level | 0.716 | 0.569-0.835 | 2.144^c^ | 0.032 |
| ^a^ Nomogram vs. PPF radiomics signature in distinguishing the positive pathological response | | | | |
| ^b^ Nomogram vs. Intratumoral radiomics signature in distinguishing the positive pathological response | | | | |
| ^c^ Nomogram vs. PSA level in distinguishing the positive pathological response  PPF, periprostatic fat; ROC, receiver operating characteristic curve; AUC, area under the receiver operating characteristic curve; CI, confidence interval | | | | |
